# Phase separation and pathologic transitions of RNP condensates in neurons: implications for amyotrophic lateral sclerosis, frontotemporal dementia and other neurodegenerative disorders

**DOI:** 10.3389/fnmol.2023.1242925

**Published:** 2023-09-01

**Authors:** Aditi Naskar, Asima Nayak, Muthu Raj Salaikumaran, Sonali S. Vishal, Pallavi P. Gopal

**Affiliations:** ^1^Department of Pathology, Yale School of Medicine, New Haven, CT, United States; ^2^Program in Cellular Neuroscience, Neurodegeneration, and Repair, Yale School of Medicine, New Haven, CT, United States

**Keywords:** neurodegenerative disease, RNA-binding proteins, aggregation, phase separation, biomolecular condensates

## Abstract

Liquid–liquid phase separation results in the formation of dynamic biomolecular condensates, also known as membrane-less organelles, that allow for the assembly of functional compartments and higher order structures within cells. Multivalent, reversible interactions between RNA-binding proteins (RBPs), including FUS, TDP-43, and hnRNPA1, and/or RNA (e.g., RBP-RBP, RBP-RNA, RNA-RNA), result in the formation of ribonucleoprotein (RNP) condensates, which are critical for RNA processing, mRNA transport, stability, stress granule assembly, and translation. Stress granules, neuronal transport granules, and processing bodies are examples of cytoplasmic RNP condensates, while the nucleolus and Cajal bodies are representative nuclear RNP condensates. In neurons, RNP condensates promote long-range mRNA transport and local translation in the dendrites and axon, and are essential for spatiotemporal regulation of gene expression, axonal integrity and synaptic function. Mutations of RBPs and/or pathologic mislocalization and aggregation of RBPs are hallmarks of several neurodegenerative diseases, including amyotrophic lateral sclerosis (ALS), frontotemporal dementia (FTD), and Alzheimer’s disease. ALS/FTD-linked mutations of RBPs alter the strength and reversibility of multivalent interactions with other RBPs and RNAs, resulting in aberrant phase transitions. These aberrant RNP condensates have detrimental functional consequences on mRNA stability, localization, and translation, and ultimately lead to compromised axonal integrity and synaptic function in disease. Pathogenic protein aggregation is dependent on various factors, and aberrant dynamically arrested RNP condensates may serve as an initial nucleation step for pathologic aggregate formation. Recent studies have focused on identifying mechanisms by which neurons resolve phase transitioned condensates to prevent the formation of pathogenic inclusions/aggregates. The present review focuses on the phase separation of neurodegenerative disease-linked RBPs, physiological functions of RNP condensates, and the pathologic role of aberrant phase transitions in neurodegenerative disease, particularly ALS/FTD. We also examine cellular mechanisms that contribute to the resolution of aberrant condensates in neurons, and potential therapeutic approaches to resolve aberrantly phase transitioned condensates at a molecular level.

## Introduction

Neurons are large, highly polarized cells that require precise spatial and temporal organization of gene expression, particularly in the axon and dendrites, in order to regulate synaptic structure and function. A growing body of evidence suggests the physical process of liquid–liquid phase separation (LLPS) allows for biological macromolecules, such as proteins and nucleic acids, to self-assemble into higher order structures within cells, including in neurons. Post-transcriptional control of RNA is accomplished by ribonucleoprotein (RNP) granules, dynamic membrane-less cellular compartments enriched in RNA and RNA-binding proteins (RBPs), which often form through LLPS (Banani et al., 2017; Yang et al., 2020). These phase separated RNP condensates include nucleoli, Cajal bodies, processing bodies, stress granules, and neuronal RNP transport granules, and each regulates distinct aspects of the RNA lifecycle, from ribonucleoprotein biogenesis, to RNA splicing, mRNA stability, transport, and translation. Strikingly, many of the RBPs involved in LLPS have been implicated in the pathogenesis of amyotrophic lateral sclerosis (ALS) and frontotemporal dementia (FTD), fatal neurodegenerative diseases with distinct but overlapping clinical, genetic and pathologic features (Ling et al., 2013).

ALS, also known as Lou Gehrig’s disease, is characterized by the selective loss of motor neurons in the brain and spinal cord and progressive muscle atrophy (Hardiman et al., 2017). In contrast, FTD is defined by neurocognitive decline and changes in social behavior, personality and language due to degeneration of the frontal and temporal cortex (Van Mossevelde et al., 2018). As many as 50% of ALS patients also develop cognitive impairment, and motor deficits are relatively common in FTD patients, underscoring the fact that these disorders exist on a clinical disease continuum (Lomen-Hoerth et al., 2002; Baizabal-Carvallo and Jankovic, 2016). While ~90% of ALS/FTD cases are “sporadic” and present without a clear family history, the remaining 5–10% of cases are associated with familial disease. Inherited and rare sporadic cases of ALS/FTD have been linked to mutations in ~40 genes, many of which encode RBPs, including transactive DNA response binding protein of 43 kDa (TDP-43) and fused in sarcoma (FUS) (Sreedharan et al., 2008; Kwiatkowski et al., 2009; Vance et al., 2009; Kim G. et al., 2020). The vast majority (>95%) of ALS and about half of FTD patients also share a common molecular histopathology, characterized by the mislocalization and aggregation of TDP-43 in the affected cells (Neumann et al., 2006; Lee et al., 2011).

**Figure 1:**
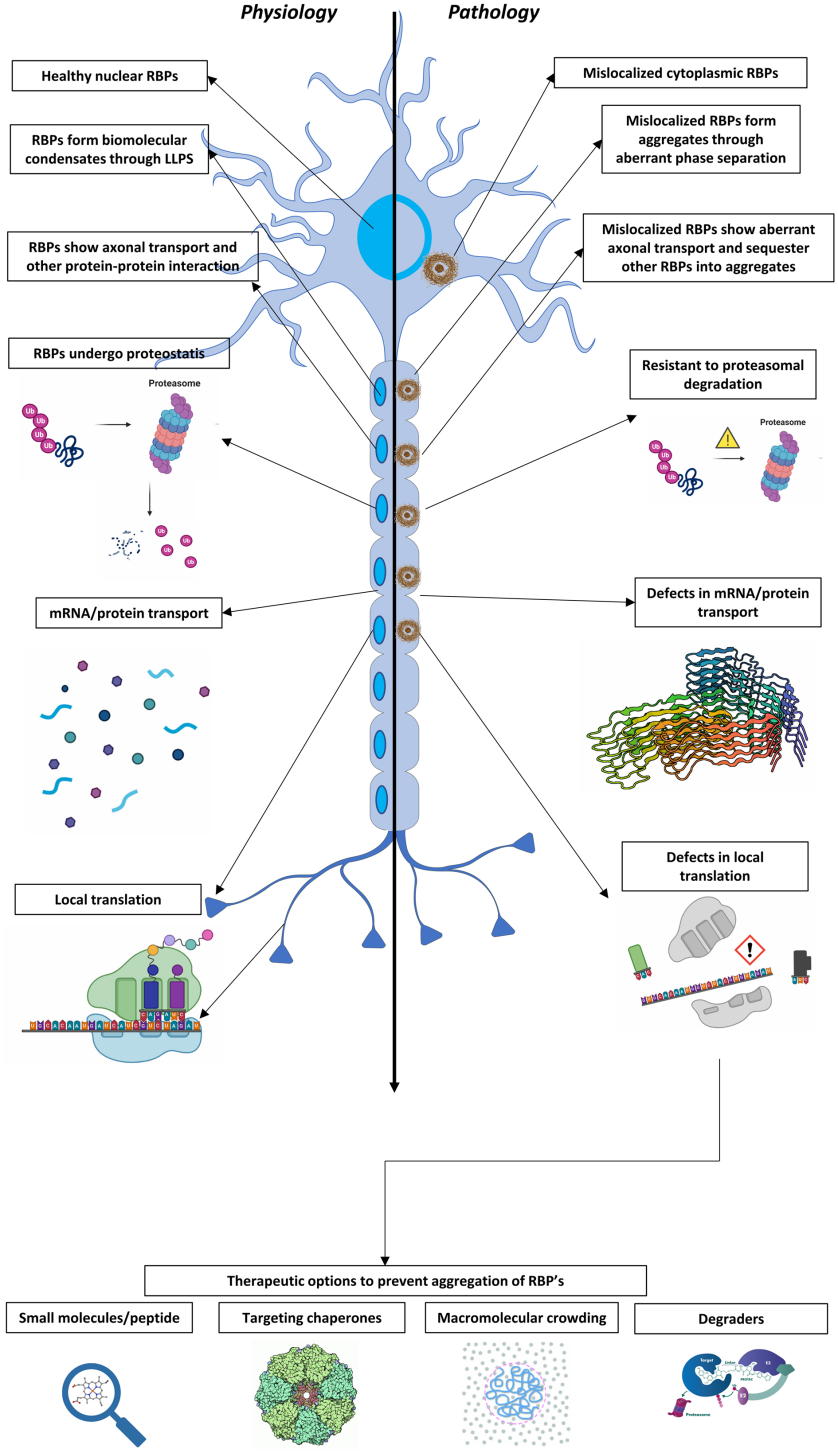
A schematic representation depicting a healthy neuron (left) and an ALS/FTD-affected neuron (right) along with the major differences in RBPs and RNP condensates under physiologic and pathologic conditions. 1. Physiological RBPs are found predominantly in the nucleus whereas RBPs are mislocalized to cytoplasm in disease. 2. Resistance to proteasomal degradation: Aggregates composed of RBPs in affected neurons show increased resistance to degradation by the cellular proteasome system. 3. Defects in mRNA/protein transport: There is impaired stability and transport of mRNA. 4. Defects in local translation: Affected neurons show deficiencies in local translation. The bottom of the figure represents several therapeutic options to target neurodegenerative diseases aimed to resolve aberrant RNP condensates and prevent the aggregation of RBPs in the affected neurons.

In this review, we specifically focus on the role of LLPS in cellular organization into liquid-like compartments, which offer advantages to cells and drive several aspects of neuronal cell biology, but also likely contribute to disease. We outline evidence that neurodegenerative disease-linked mutations of RBPs lead to aberrant phase separation, altered condensate dynamics and biophysical properties, and disruption of critical cellular and molecular functions (Figure 1). Although we largely concentrate on aberrant LLPS in ALS/FTD, we also discuss data that suggest aberrant phase separation plays a role in pathological protein aggregation in Alzheimer’s disease (AD) and Parkinson’s disease (PD). Finally, we discuss cellular mechanisms to combat aberrant LLPS as well as potential therapeutic approaches.

## Liquid–liquid phase separation: a mechanism for the assembly of biomolecular condensates

Phase separation is a fundamental thermodynamic process in which two (or more) macromolecules preferentially interact with one another and demix from a homogeneous solution to form distinct phases (Banani et al., 2017). In cells, multivalent low-affinity interactions between biomolecules present at high local concentrations (e.g., RNA–RNA, RNA-protein, protein–protein) drive the condensation of proteins, RNA, and membrane-bound organelles into liquid-like foci, allowing for the formation of biomolecular condensates, also known as membrane-less organelles (MLOs) (Banani et al., 2017; Ma and Mayr, 2018). In neurons, LLPS facilitates the assembly of specialized biomolecular condensates including RNP condensates (e.g., neuronal RNP transport granules, discussed below), membrane-associated higher-order assemblies at the synapse (e.g., pre-synaptic active zone) and synaptic vesicle condensates (Milovanovic et al., 2018; Liao et al., 2019; Wu et al., 2019; McDonald et al., 2020). The phenomenon of LLPS is governed by the balance between interaction energies of biomolecules (i.e., enthalpy) and entropy of the system, which can be modulated by temperature, molecular crowding, solvent conditions, macromolecule concentrations and composition (Hyman et al., 2014; Nott et al., 2016). These multivalent and low-affinity interactions promote a dynamic exchange of components within the biomolecular condensate and the surrounding environment (Banani et al., 2017).

**Figure 2:**
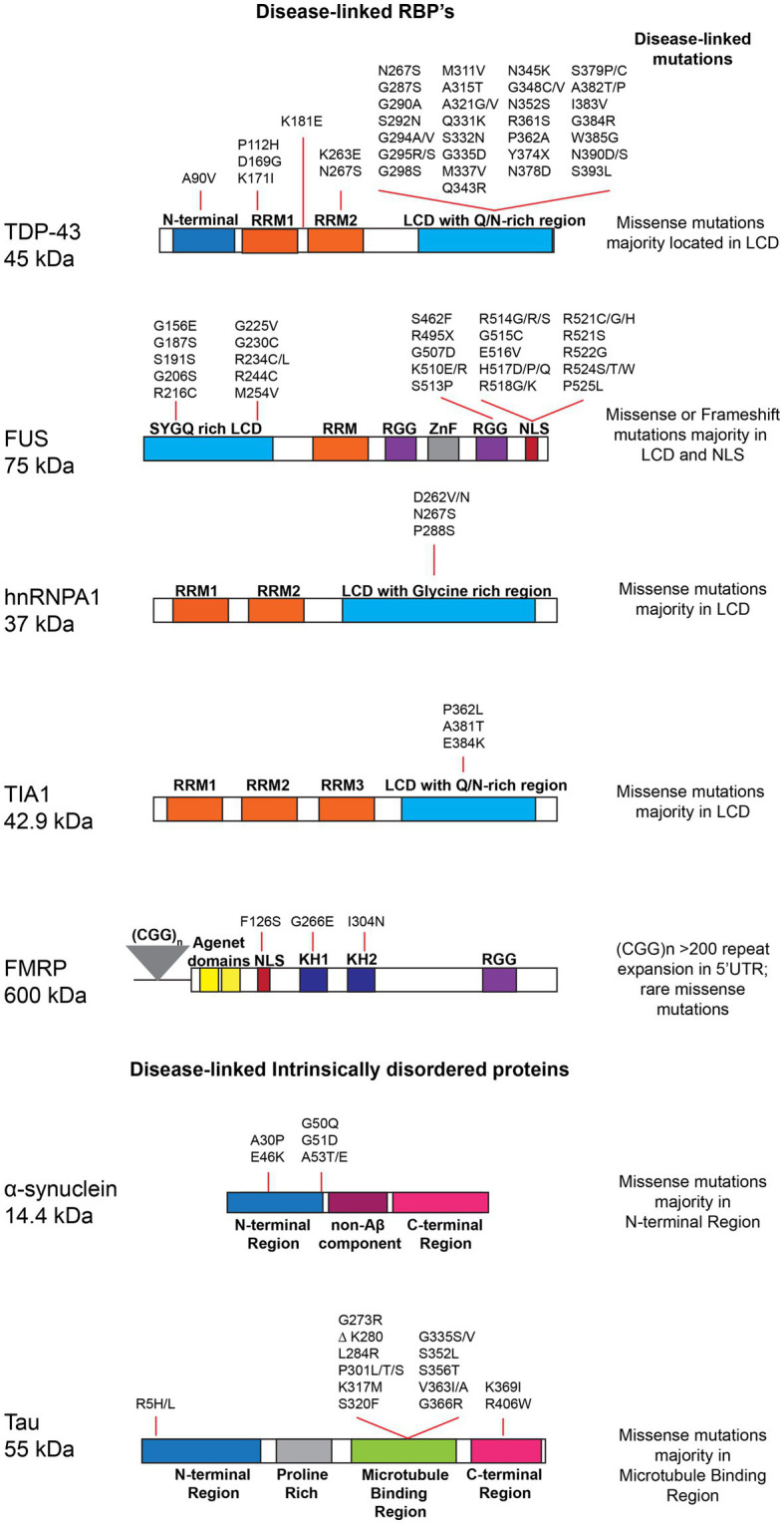
Schematic highlighting RNA-binding proteins (RBPs) and intrinsically disordered proteins (IDPs), domain structure and disease-linked mutations: TAR DNA-binding protein 43 (TDP-43 - Uniprot Q13148), Fused in sarcoma (FUS - Uniprot P35637), Heterogeneous nuclear ribonucleoprotein A1 (hnRNPA1 Uniprot P09651), T-cell intracellular antigen 1 (TIA1 - Uniprot P31483), Fragile X mental retardation protein (FMRP – Uniprot Q06787), α-Synuclein (Alpha-synuclein – Uniprot P37840), and Microtubule-associated protein tau (Tau - Uniprot P10636-5). The major domains for each protein and disease-linked mutations retrieved from Uniprot, are depicted. RNA recognition motif (RRM); Low complexity domain (LCD); Zinc finger (ZnF); Tyrosine- and glycine-rich region (YG-box); Frameshift (FS); Loss of function (LOF); Nuclear localization signal (NLS); hnRNP K protein homology (KH); arginine-glycine–glycine box (RGG); Non-Amyloid-beta component (NAC) region, Microtubule binding region (MTBR).

Biomolecular condensates are often composed of proteins with modular interaction domains or intrinsically disordered regions (IDRs) (Li et al., 2012; Lin et al., 2015). IDRs lack stable tertiary structures, can adopt multiple conformations and frequently contain repetitive low-complexity sequences (Banani et al., 2017). For example, the IDRs of many RBPs are referred to as low complexity domains (LCDs) because they are enriched in polar (e.g., glycine, serine, glutamine, asparagine), aromatic (e.g., tryptophan and tyrosine), and/or charged residues (Kato et al., 2012; Mittag and Parker, 2018; Figure 2). Several studies suggest these repeated sequence elements drive multivalent, low-affinity electrostatic, dipolar or hydrophobic interactions between aromatic, polar and/or charged residues in the IDRs of RBPs, and thus promote LLPS *in vitro* and condensate formation in cells (Gilks et al., 2004; Decker et al., 2007; Kato et al., 2012; Brangwynne et al., 2015; Nott et al., 2015; Lin et al., 2017; Wang et al., 2018). In addition, purified RBPs with IDRs, including many disease associated RBPs such as FUS (Han et al., 2012; Patel et al., 2015), hnRNPA1 (Kim et al., 2013; Molliex et al., 2015), TDP-43 (Conicella et al., 2016; Li et al., 2018; McGurk et al., 2018), TIA-1 (Mackenzie et al., 2017), and FMRP (Kim et al., 2019; Tsang et al., 2019), have been shown to undergo LLPS *in vitro*. Furthermore, multivalent interactions between aromatic and/or charged residues in the IDRs and RNA-binding domains of RBPs as well as RNA–RNA and RBP-RNA interactions also play an important role in driving phase separation (Elbaum-Garfinkle et al., 2015; Lin et al., 2015; Molliex et al., 2015; Zhang H. et al., 2015; Wang et al., 2018; Grese et al., 2021). In cells, the formation of RNP condensates integrates both specific protein-RNA and protein–protein interactions along with the relatively weak non-specific IDR interactions that drive LLPS *in vitro* (Mittag and Parker, 2018). However, the relative contributions of these different types of interactions for RNP condensate formation in cells is not well understood but is likely context-specific and involves post-translational modifications, for example, that alter weak multivalent interactions between IDRs (Sanders et al., 2020; Yang et al., 2020). Additional research is required to elucidate the complex network of interactions driving RNP condensate assembly and regulation in neurons and other disease-relevant cells.

## Functions of physiological RNP condensates in neurons

In cells, biomolecular condensates serve to accelerate biochemical reactions by increasing local enzyme and substrate concentrations, or alternatively, may modulate reactions by sequestering or buffering condensate components from the cytoplasm (Strulson et al., 2012; Banjade and Rosen, 2014; Tatomer et al., 2016). RNP condensates in particular serve as temporary reservoirs for specific mRNAs and RBPs, where they store, stabilize and/or transport translationally repressed mRNA, ultimately contributing to the function of synapses, as well as the architecture and organization of neurons (Kim N. Y. et al., 2020; Nedelsky and Taylor, 2022).

Neuronal transport granules, stress granules, and processing (P)-bodies are distinct but related cytoplasmic RNP condensates that have vital functions in the post-transcriptional processing of RNA, as well as mRNA subcellular localization, translation, storage, and turnover (Kiebler and Bassell, 2006; Buchan and Parker, 2009; Donlin-Asp et al., 2017). Stress granules (SGs) are transient RNP condensates that contain translationally stalled mRNAs, pre-initiation factors, ribosomal components and specific RBPs (Buchan and Parker, 2009; Kedersha et al., 2013; Jain et al., 2016). SGs emerge in response to various forms of cellular stress such as viral infections, heat shock, and oxidative stress (Anderson and Kedersha, 2009). SGs prevent the degradation of mRNAs that have undergone translation arrest, by storing them alongside the necessary translation initiation factors and RBPs. During stress, SGs also act as signaling hubs and work to suppress the production of new proteins that might be unnecessary (those involved with cellular growth) or detrimental (those associated with apoptosis or cytotoxicity) to promote cell survival (Kedersha et al., 2013; Wippich et al., 2013; Kanda et al., 2021). Independent of stress, depletion of SG scaffolding protein G3BP1 alters intracellular calcium homeostasis and results in abnormal synaptic plasticity in neurons (Martin et al., 2013; Yang et al., 2020). Recent research also suggests that cellular aging impacts SG assembly and dynamics of primary motor neurons, though the relevance of these findings for function *in vivo* remains to be elucidated (Khalfallah et al., 2018).

P-bodies are involved in mRNA breakdown, storage, and translational repression (Adeli, 2011). They contain a wide variety of enzymes involved in mRNA degradation, including decapping enzymes, exonucleases, and RISC (RNA-induced silencing complex) components (Parker and Sheth, 2007). P-bodies may regulate synaptic plasticity, neuronal development, and stress response, but further research is needed to completely understand the functions of P-bodies in neurons (Nonhoff et al., 2007). Dysregulation of P-bodies has been linked to a number of neurodegenerative disorders, including AD and ALS (Wootz et al., 2013; Wen et al., 2014; Barmada et al., 2015).

Neuronal transport granules facilitate the long-distance transport of mRNAs from the cell body to the axon and dendrites, enabling local translation and activity-dependent regulation of protein synthesis in distal neuronal compartments and at the synapse (Ainger et al., 1993; Krichevsky and Kosik, 2001; Kanai et al., 2004; Willis et al., 2005). Maintaining local translation is crucial for synaptic function, regulating dendritic spine morphology, and modulating long-term potentiation (LTP) and long-term depression (LTD) (Sutton and Schuman, 2006; Holt and Schuman, 2013). In neurons, mRNA transport and local translation are tightly regulated by RBPs. In response to certain cues, the mRNAs are released from the RNP granules and translated by local ribosomes, resulting in the synthesis of proteins necessary for synaptic function, plasticity, and neuronal homeostasis (Steward and Schuman, 2003; Bramham and Wells, 2007; Buxbaum et al., 2014). For instance FMRP is believed to regulate the local storage and translation of mRNAs at synapses, where it binds to many mRNAs in neurons (Siomi et al., 1993; Brown et al., 2001; Hagerman et al., 2001; Huber et al., 2002; Antar et al., 2004; Muddashetty et al., 2007; Darnell et al., 2011; Nelson et al., 2013; Alpatov et al., 2014; Pasciuto and Bagni, 2014; Hu et al., 2015; Myrick et al., 2015; D'Souza et al., 2018; Kute et al., 2019) forming RNP complexes that are then transported along microtubules by kinesin and dynein motor proteins (Antar et al., 2005, 2006; Dictenberg et al., 2008). Similar to FMRP, ALS/FTD-linked RBPs such as FUS and TDP-43, are components of neuronal transport granules (Kanai et al., 2004; Elvira et al., 2006; Fallini et al., 2012; Liu-Yesucevitz et al., 2014). In addition to their critical roles in the nucleus, TDP-43 and FUS also regulate the trafficking of specific mRNAs in the dendrites and axon, particularly transcripts encoding cytoskeletal-related proteins (Fujii et al., 2005; Fujii and Takumi, 2005; Strong et al., 2007; Alami et al., 2014; Coyne et al., 2014; Chu et al., 2019). The pool of mRNAs stored in neuronal transport granules must be readily available for translation in response to signals from the cell as reported in the case of Staufen proteins (STAU1 and STAU2) (Duchaîne et al., 2002; Graber et al., 2013). Thus, neuronal transport granules play a critical role in the storage and regulated translation of mRNA, as well as its localization and stability (Buxbaum et al., 2014, 2015).

## Aberrant phase separation in neurodegenerative disease

Defects in RNA metabolism characterize a large proportion of neurodevelopmental and neurodegenerative disorders (Prashad and Gopal, 2021). In fact, there is strong human genetic evidence that mutations of numerous RBPs are linked to ALS and FTD (Ling et al., 2013). Notably, many of these ALS/FTD-linked mutations cluster in the low complexity domains of RBPs and/or disrupt their nuclear localization (Figure 2), raising the possibility that these mutations disturb the delicate balance of low affinity interactions driving physiological phase separation, alter subcellular localization of RBPs, and/or increase the propensity for pathological aggregation (Buratti, 2015; Nedelsky and Taylor, 2022). Indeed, several *in vitro* studies of purified ALS/FTD-linked mutant forms of TDP-43, FUS, hnRNPA1, and TIA-1 show that they exhibit aberrant phase separation, accelerate the formation of solid or gel-like structures and lead to thioflavin positive fibrillar aggregates (Molliex et al., 2015; Patel et al., 2015; Conicella et al., 2016; Mackenzie et al., 2017; McGurk et al., 2018). Moreover, ALS-linked mutations of RBPs, have been shown to perturb post-transcriptional RNA processing, mRNA trafficking and local translation, stress granule formation/dissolution kinetics, and/or mRNA stability in addition to altering RNP condensate behavior *in vivo* and in cellular disease models (Kim et al., 2013; Coyne et al., 2015; Murakami et al., 2015; Gopal et al., 2017; Fratta et al., 2018; Wolozin and Ivanov, 2019; Hallegger et al., 2021).

While it is difficult to distinguish functional defects induced by the RBP mutation itself vs. compromised function due to aberrantly phase separated RNP condensates, it has been suggested that aberrant phase separation may offer a unifying explanation for the plethora of functional defects in RNA regulation observed with pathological mutations of RBPs (Nedelsky and Taylor, 2022). In addition, examples of aberrant condensates associated with pathologic post-translational modifications of wild type RBPs or other disordered proteins such as tau, suggest that aberrant phase separation may also be relevant in the setting of sporadic neurodegenerative disease (Cohen et al., 2015; Wegmann et al., 2018; Yu et al., 2021; Lu et al., 2022). Here we consider potential mechanisms of how aberrant phase separation may be detrimental to function and evaluate the data indicating that aberrant LLPS contributes to the formation of pathological aggregates in neurodegenerative disease (Patel et al., 2015; Alberti et al., 2019). Table 1 provides a summary of notable RBPs and intrinsically disordered proteins linked to aberrant phase separation in ALS/FTD and other neurodegenerative diseases.

**Table 1:** Neurodegenerative disease-linked RBPs and Intrinsically disordered proteins.

	Major domains	Function	Associated diseases	Interacting partners	Key references
Disease-linked RBPs					
TDP-43 (45 kDa)	N-terminal, RRM1, RRM2, C-terminal low complexity domain	DNA/RNA binding protein that regulates RNA metabolism and splicing, stress granule dynamics	ALS, FTD	hnRNPA1, FUS, TIA1, Matrin 3	Kitchell et al. (1978), Liu-Yesucevitz et al. (2010), Ayala et al. (2011), Salton et al. (2011), Ling et al. (2013), Honda et al. (2015), Zhang K. et al. (2015), Lewis-Smith et al. (2016), Mackenzie et al. (2017), Wang et al. (2018), Schmidt et al. (2019), and Vishal et al. (2022)
FUS (75 kDa)	QGSY (glutamine-glycine-serine-tyrosine)-rich region, RRM, Zinc finger domain, NLS, RGG, C-terminal domain	DNA/RNA binding protein that regulates RNA metabolism and splicing	ALS, FTD	TDP-43, hnRNPA1, SMN, EWSR1, TAF15, Transportin-1, Ataxin-1	Kwiatkowski et al. (2009), Vance et al. (2009), Lagier-Tourenne et al. (2010), Deng et al. (2011), Ju et al. (2011), Deng et al. (2014), and Murakami et al. (2015)
hnRNPA1 (37 kDa)	RBDs with Glycine rich domains and C-terminal domain with an M9 Transport Receptor Binding Domain	DNA/RNA binding protein that regulates RNA metabolism and splicing	ALS, FTD	FUS, TDP-43, Matrin 3, Ataxin-2, Transportin-1, Caprin-1	Donev et al. (2007), Choi et al. (2012), Ishigaki et al. (2012), Kim et al. (2013), Chen et al. (2014), Honda et al. (2015), Xue et al. (2020), and Beijer et al. (2021)
TIA1 (43 kDa)	RRM1,2,3 and LCD (Low complexity domain)	DNA/RNA binding protein that regulates RNA metabolism and splicing, involved stress granule formation	ALS, FTD	hnRNPA1, FUS, TDP-43, Caprin-1, G3BP1, G3BP2, PABP1, PABP4	Kedersha and Anderson (2002), Markmiller et al. (2018), Jiang et al. (2019), Fritzsching et al. (2020), and Loughlin et al. (2021)
MATR3 (95 kDa)	RRM1,2, NES (nuclear export signal), NLS (Nuclear localization signal)	DNA/RNA binding protein that regulates RNA metabolism and splicing	ALS, FTD	TDP-43, hnRNPA1, FUS, TAF15, EWSR1, Transportin-1, HSP70, HSP90	Monahan et al. (2016), Boehringer et al. (2017), Ramesh et al. (2020), Xue et al. (2020), and Malik and Barmada (2021)
FMRP (600 kDa)	Tudor, KH0 domains, two K Homology (KH) domains and one RGG domain in C-terminal domain	Translation Regulation, RNA transport, chromatin remodeling, ion channel stability, and maintaining synaptic development	Fragile X Syndrome / Fragile X-associated Tremor/Ataxia Syndrome (FXTAS)	CYFIP1, FXR1, FXR2, and various mRNAs.	Siomi et al. (1993), Brown et al. (2001), Hagerman et al. (2001), Huber et al. (2002), Antar et al. (2004), Muddashetty et al. (2007), Darnell et al. (2011), Nelson et al. (2013), Alpatov et al. (2014), Pasciuto and Bagni (2014), Hu et al. (2015), Myrick et al. (2015), D'Souza et al. (2018), and Kute et al. (2019)
Disease-linked Intrinsically disordered proteins					
α-synuclein (14.4 kDa)	N-terminal, Non-Amyloid-beta component (NAC) region, C-terminal domain	Synaptic function	Parkinson’s Disease	Tubulin, parkin, phospholipasesD, Tau, Dopamine receptors	Ostrerova et al. (1999), McLean et al. (2000), Lee F. J. et al. (2001), Chen et al. (2007), Yu et al. (2007), Kawahara et al. (2008), Gorbatyuk et al. (2010), Venda et al. (2010), and Chen et al. (2013)
Tau (33–55 kDa)	N-terminal, Proline-rich region, microtubule binding region, C-terminal	Stabilizes neuronal microtubules under normal physiological conditions	ALS, FTD, parkinsonism-17	TIA1, TDP-43, MAP2K7, CDC37	Zhu et al. (2001), Lee V. M. et al. (2001), Avila et al. (2004), Jinwal et al. (2011), Vanderweyde et al. (2016), Gao et al. (2018), Chornenkyy et al. (2019), and Paterno et al. (2022)

### Loss of RNP condensate liquid-like properties impairs mRNA transport

A growing body of evidence supports the view that aberrantly phase separated RNP condensates composed of mutant RBPs result in loss of dynamic liquid-like biophysical properties, which compromises their RNA regulatory functions in neurons. For example, ALS-linked mutations of FUS and TDP-43 promote hardening of neuronal RNP condensates (Murakami et al., 2015; Gopal et al., 2017). Neuronal RNP transport granules containing mutant TDP-43 display impaired transport in dendrites (Liu-Yesucevitz et al., 2014) and along the axon of primary cortical neurons or ALS patient iPSC-derived neurons (Alami et al., 2014; Gopal et al., 2017) as well as increased viscosity and loss of liquid-like properties (Gopal et al., 2017; Vishal et al., 2022). Furthermore, ALS-linked mutations of TDP-43 disrupt axonal transport and/or stability of *Nefl* and *futsch/MAP1B* mRNA (Alami et al., 2014; Coyne et al., 2014). Similarly, FUS mutations disrupt axonal transport of *Dynll2* and *Kif5b* mRNA in primary hippocampal neurons and localization of *Ddr2* mRNA to the cell periphery (Yasuda et al., 2017). Thus, defects in neuronal RNP transport granule dynamics impair mRNA trafficking and localization of critical transcripts to the axon, dendrites, and neuromuscular junction (NMJ), which may in turn compromise synaptic and NMJ homeostasis and trigger axonal degeneration.

### Sequestration of mRNAs and RBPs dysregulate local and global translation

Several studies suggest that another key consequence of dynamically arrested, aberrant RNP condensates and is the sequestration of mRNA and RBPs resulting in defects in multiple cellular functions, most prominently translation (Kim N. Y. et al., 2020). Missense mutations of arginine residues in FUS (i.e., R244C, R216G, R521G) sequester wild type (WT) FUS and disrupt WT FUS-RNA droplet fluidity (Rhine et al., 2020). Moreover, ALS-linked mutant FUS mislocalizes to the cytoplasm, sequesters FMRP into FUS-FMRP condensates in an RNA-dependent manner, and suppresses translation in motor neurons (Birsa et al., 2021). Additional support for translation dysregulation associated with aberrant FUS condensates comes from a proteomic analysis of ALS-mutant FUS cytoplasmic inclusions, which reveals an enrichment of stalled ribosomal complexes and factors promoting nonsense mediated decay, compared to wild type FUS (Kamelgarn et al., 2018). Spinal cords of FUS mutant [R521C and R521H] transgenic mice show depletion of mRNAs encoding ion channels, transporters and ribosomal proteins, and furthermore, these FUS mutations inhibit local translation in axons by inducing stress mediated phosphorylation of eIF2α (Lopez-Erauskin et al., 2018). In other instances, mutant FUS binds the 3’UTR of transcripts, such as the *ELAVL4* mRNA, and increases its translation, but also sequesters ELAVL4 (HuD) and other RBPs within stiff, gel-like condensates (De Santis et al., 2019).

There is also evidence that pathologic C-terminal fragments of wild type TDP-43 and mislocalized hyper-phosphorylated wild type TDP-43 are associated with aberrant RNP condensates and aggregate formation (Igaz et al., 2009; Altman et al., 2021). For example, pathologic C-terminal fragments of wild type TDP-43 sequester RBPs such as TIA-1, PABPC1, hnRNPA2/B1 in a RNA dependent manner (Jiang et al., 2022). Moreover, aberrant phosphorylated TDP-43 condensates in the axon of motor neurons sequester G3BP and nuclear encoded mitochondrial mRNA *Cox4i1* and *ATP5A1,* and impair local protein synthesis of nuclear encoded mitochondrial genes (Altman et al., 2021). Aside from inhibiting translation, aberrant condensates are also likely to sequester key proteins in the proteosome and autophagy pathways, compromising proteostasis and organelle quality control mechanisms (discussed below) (Wakabayashi et al., 2002; Kuusisto et al., 2003; Shao et al., 2022).

### Aberrant condensates and transition to pathological fibrillar aggregates

In addition to functional defects in RNA regulation associated with aberrantly phase separated condensates, evidence suggests that in some cases, these dynamically arrested condensates can give rise to fibrillar protein aggregates that are the pathologic hallmarks of ALS/FTD and other neurodegenerative diseases (Ramaswami et al., 2013). For example, aspartate to valine mutations in the prion-like domain of hnRNPA1 family of proteins accelerate the formation of fibrils and of cytoplasmic inclusions leading to multisystem proteinopathy and ALS (Kim et al., 2013). Similarly, ALS-linked mutations of FUS promote the molecular aging of liquid FUS condensates and conversion to fibrillar aggregates, leading to formation of cytoplasmic inclusions (Patel et al., 2015; Birsa et al., 2021). PD-linked mutations of α-Synuclein, which is a synaptic protein normally found at axon terminals, promote LLPS and maturation of α-Synuclein condensates, resulting in the formation of perinuclear cytoplasmic α-Synuclein aggregates (Ray et al., 2020). On the other hand, mutations in the alpha-helical region of TDP-43’s low complexity domain disrupt LLPS but also promote aggregation (Buratti, 2015; Che et al., 2015; Conicella et al., 2016). In addition, under stress conditions in cells, cytoplasmic TDP-43 aggregates can form independently and are distinct from stress granules (Gasset-Rosa et al., 2019).

Apart from aberrant condensates associated with neurodegenerative disease-linked mutations, post-translational modifications of wild type RBPs or other IDPs such as tau, may also result in aberrant condensates or aggregates. Pathologic TDP-43 aggregates found in the majority of sporadic ALS cases display numerous post-translational modifications of wild type TDP-43, including phosphorylation, ubiquitination, cysteine oxidation, acetylation, and truncation (Neumann et al., 2006; Buratti, 2015; Cohen et al., 2015; Arseni et al., 2022). Acetylation of TDP-43 in particular promotes the formation of nuclear TDP-43 condensates but also alters TDP-43 solubility and promotes aggregation (Cohen et al., 2015). A similar observation is seen in the case of phosphorylated wild type Tau protein, which forms phase separated phospho-tau droplets that go on to initiate Tau aggregates. Furthermore, high molecular weight Tau isolated from human AD brain tissue also undergoes LLPS and aggregation (Wegmann et al., 2018).

### Role of persistent SGs in neurodegenerative disease

Due to their complex structure and post-mitotic nature, neurons experience various forms of acute and chronic stress over their long life-span. To overcome oxidative or proteo-stress conditions for instance, neurons depend on an efficiently regulated SG response (Wolozin and Ivanov, 2019). Dysregulation of SG formation/dissolution kinetics and persistence of SGs has been suggested as the first step toward aberrant phase separation and formation of insoluble protein aggregates in ALS/FTD (Li et al., 2013; Mackenzie et al., 2017). Indeed, a subset of TDP-43 positive aggregates and phosphorylated-tau in ALS/FTD and AD patient brain tissue, respectively, contain SG markers (Liu-Yesucevitz et al., 2010; Bentmann et al., 2012; Maziuk et al., 2018; Wolozin and Ivanov, 2019). For example, TDP-43 aggregates in the spinal cord of ALS patients are positive for TIA-1, PABP-1, or other canonical SG markers (Liu-Yesucevitz et al., 2010; Bentmann et al., 2012). However, in the hippocampus of FTD patients with TDP-43 pathology, SG RBPs show more inconsistent overlap with aggregates (Bentmann et al., 2012; Aulas and Vande Velde, 2015), and mature neurofibrillary tangles in AD patient tissue do not colocalize with SG markers (Maziuk et al., 2018). These findings may reflect potential differences in SG biology of motor neurons compared to hippocampal and cortical neurons, and/or perhaps highlight differences in the role of persistent SGs in ALS vs. FTD and AD. Furthermore, as mentioned above, there is also evidence that under cellular stress paradigms, cytoplasmic TDP-43 aggregates which form are distinct from SGs, suggesting that there are both SG-dependent and independent mechanisms driving TDP-43 pathology (Aulas and Vande Velde, 2015; Gasset-Rosa et al., 2019). Additional *in vivo* evidence that demonstrates the transformation of persistent SGs to mature pathologic aggregates is needed in longitudinal ALS/FTD and AD disease models in order to elucidate the mechanistic role of SGs in these diseases.

Taken together, the literature suggests that while pathologic aggregation of RBPs and other intrinsically disordered proteins in neurodegenerative disease may occur via aberrant phase separation and persistence of SGs in some instances, protein misfolding and aggregation in disease also occurs independent of LLPS. Regardless of the mechanism leading to pathologic aggregates in disease, cells have evolved several mechanisms to prevent aggregation and/or resolve aberrantly phase separated condensates.

## Cellular mechanisms to overcome aberrant phase separation

For the majority of patients with ALS/FTD or other neurodegenerative disease who do not have highly penetrant mutations in RBPs or other IDPs such as α-synuclein, it has been suggested that aging and cellular stress, induce changes in protein conformation or stability that may promote aberrant phase separation and/or aggregation. Indeed, human genetics data strongly implicate defects in lysosomal trafficking, autophagy, and protein quality control mechanisms in ALS/FTD, AD and PD, indicating that mutations in these pathways may also trigger neurodegenerative disease (Rogaeva et al., 2007; Caglayan et al., 2014; Bras et al., 2015; Guerreiro et al., 2015). Under stress conditions, proteins may adopt altered conformations that expose hydrophobic regions, which can promote the formation of solid-gel like aggregates (Gasset-Rosa et al., 2019). Therefore, to combat aberrantly phase separated condensates, cells have evolved mechanisms to regulate LLPS as well as quality control mechanisms (Prasad et al., 2010). Post-translational modifications, such as phosphorylation, ubiquitination, and acetylation, can also modulate the phase behavior of proteins by altering their charge or conformation, ultimately affecting their ability to undergo aberrant phase separation (Hofweber et al., 2018; Maziuk et al., 2018). In this section we describe our current understanding of these homeostatic mechanisms and their neuroprotective functions.

### Role of lysosomes

Biomolecular condensates are known to interact with and recruit membrane-bound organelles like endoplasmic reticulum and lysosomes (Ma and Mayr, 2018; Liao et al., 2019; Zhou, 2022). For example, Liao and colleagues showed that Annexin A11 (ANXA11) serves as an adaptor protein that tethers RNP granules and lysosomes, allowing RNP condensates to hitchhike on lysosomes (Liao et al., 2019). Whether membrane-bound organelles like lysosomes are recruited by cells to clear protein aggregates is poorly understood. One of the hallmark features of ALS is the aggregation of FUS upon cellular stress. FUS aggregates are reported to recruit lysosomes (Trnka et al., 2021). This observation suggests that lysosomes may be able to sense aberrantly phase separated condensates and amass more near solid-gel like aggregates rather than fluid condensates. Additional studies are needed to determine whether this is a potential mechanism to clear aggregates and aberrantly phase separated condensates.

### Role of ubiquitin proteasome system and autophagy

The ubiquitin proteasome system (UPS) and autophagy are the two major proteolytic systems in the eukaryotic cells responsible for the degradation of proteins and protein aggregates. Aggregation prone proteins such as poly (Q) expansion repeats, mutant (SOD1), α-synuclein and tau can be degraded by both (UPS) and autophagy (Zheng et al., 2009). The UPS involves ubiquitination of proteins with the help of a cascade of enzymes (E1-ubiquitin activating enzyme, E2-ubiquitin conjugase, E3- ubiquitin ligase) consequently targeting them to the proteasomal machinery (Ciechanover and Schwartz, 1998). Multiple studies have shown the clearance of soluble TDP-43 (van Eersel et al., 2011) and mutant TDP-43 aggregates by the UPS (Scotter et al., 2014). Autophagy pathway is activated when the UPS cannot degrade aggregated proteins because of their failure to enter the proteasomal barrel (Verhoef et al., 2002).

Autophagy is a cellular degradation process that involves engulfing and enclosing the cytoplasmic contents, mainly the damaged cellular components, in an autophagosome preceding lysosomal degradation (Nixon, 2013). An autophagosome engulfs parts of the cytosol and fuses with a lysosome leading to autophagolysosome where its contents are broken down (Deter et al., 1967). The autophagosomes play a key role in degrading the stress granules that undergo a decelerated degradation process or that fail to disassemble (Buchan et al., 2013). According to a study, *C9orf72* repeat expansions hoard arginine methylated proteins and incorporate autophagy receptor P62 to promote elimination of stress granules by autophagy (Chitiprolu et al., 2018). Kinases like TANK-binding kinase (TBK-1) helps in autophagy mediated degradation of protein aggregates by phosphorylating autophagy adaptor p62 (Shao et al., 2022). A recent study showed *C9orf72* expansion sequesters TBK-1 into poly GA inclusions thereby diminishing TBK1’s function which in turn impedes endosomal maturation (Shao et al., 2022). Hence, kinase activity of TBK1 is important to target the clearance of aggresomes with the help of the endolysosomal system.

### Role of chaperones and nuclear import receptors (NIRs)

Aberrantly phase transitioned stress granules carrying misfolded proteins are disassembled by chaperones upon subsidence of stress. Heat shock proteins (HSPs) act as chaperones in both prokaryotes and eukaryotes, thereby promoting proteostasis and protecting from cellular stress (Lindquist and Craig, 1988). Although few HSPs are constitutively expressed while the remaining are upregulated in stressful conditions (Miller and Fort, 2018). Studies in yeast and *Drosophila* showed that the chaperones of the HSP70, HSP104 families help disaggregate (Cherkasov et al., 2013; Kroschwald et al., 2015) the misfolded proteins from SGs. In mammalian cells, chaperone HSP72 is critical for SG dissolution (Mazroui et al., 2007). Other studies have shown that HSP70, BAG3 (nucleotide-exchange factor), HSPB8 (small heat shock protein) promote SG quality control and favor the disassembly of stress granules (Minoia et al., 2014; Crippa et al., 2016; Ganassi et al., 2016). Further, ALS-associated variants of SOD-1, susceptible to misfolding, aggregate into SGs thereby interfering with the dynamic liquid like property of SG and turning them into an aberrant condensate (Das et al., 2023). After the removal of stress, chaperones like HSP27, HSP70 can avert aberrant SG formation and promote SG disassembly in SOD1 positive SGs (Mateju et al., 2017).

Physiological stress granules, if not resolved, can transform into aberrantly phase transitioned condensates. The intrinsically disordered region of FUS is prone to form aggregates and accumulates in SGs and promotes their maturation (Li et al., 2013; Patel et al., 2015). HSPB8 can decelerate FUS induced SG maturation (Boczek et al., 2021). HSP family protein variants of HSP104 are known to suppress toxicity and aggregation caused by TDP-43, FUS and α-synuclein in yeast models (Jackrel et al., 2014; Jackrel and Shorter, 2014). Moreover, synthetic HSP104 variants are capable of dissolving cytoplasmic ALS-linked FUS aggregates in mammalian cells (Yasuda et al., 2017). Mammalian HSPB1, an ATP dependent chaperone in association with other chaperones of HSP-70 family, BAG2, BAG3 and HSP40, help in disassembly of de-mixed droplets of TDP-43 in ALS upon removal of stress thereby preventing the formation of TDP-43 gel like condensates (Lu et al., 2022).

Not only chaperones, but kinases are also involved in stress granule disassembly. For example, kinases like Casein kinase 2 (CK2) cause SG disassembly by phosphorylating the SG nucleating protein G3BP1 (Reineke et al., 2017). DYRK3 (dual specificity tyrosine phosphorylation regulated kinase 3), another kinase can regulate liquid–liquid unmixing in cells. This kinase is capable of dissolving SGs by promoting phosphorylation of several RNA binding proteins (Wippich et al., 2013), as well as other MLOs, such as splicing speckles, centrosomes (Rai et al., 2018).

Various nuclear RNA-binding proteins (RBPs) like TDP-43, FUS, hnRNPA1, and hnRNPA2, upon mutation or stress can mislocalize to cytoplasm and form aggregates leading to several neurodegenerative disorders. Guo et al. (2018) demonstrated that nuclear import receptors (NIRs) can recognize nuclear localization signals (NLS) of wild type and disease linked RBPs with prion like domains, including TDP-43, FUS, and hnRNPA1/A2, and disaggregate them (Guo et al., 2018). Karyopherin-β2 acts as a molecular chaperone for RBPs with NLS containing proline and tyrosine residues (PY-NLS) like FUS, hnRNPA1and reverses their aberrant phase transitions (Fare et al., 2023) whereas Importin-α, in consort with Karyopherin-β1, reverses TDP-43 fibrillization (Guo et al., 2018).

To summarize, autophagosomes, endolysosomal systems, chaperones, NIRs and kinases are critical cellular defense mechanisms to combat aberrant phase separation. Further research is required to exploit these mechanisms to generate therapeutic treatments modalities for neurodegenerative diseases.

## Molecular therapeutic approaches targeting aberrantly phase separated condensates and aggregation of intrinsically disordered proteins in neurodegenerative disease

As discussed above, in disease states and/or during aging, aberrant phase transitions of RNP condensates may lead to the accumulation of misfolded proteins and/or the formation of pathologic aggregates over time (Tauber et al., 2020). The increasing number of examples of aberrant condensate behavior associated with cellular and molecular dysfunction in neurodegenerative disease states highlight a need to develop new therapeutic approaches for modulating biomolecular condensates as well as preventing protein aggregation.

However, because disease-linked RBPs are enriched in IDRs and lack stable, well-defined binding pockets, they have largely been considered undruggable. Furthermore, the specific structural aberrations observed in RBPs under pathological conditions can vary depending on the proteins involved, the cytoplasmic milieu, and the disease context (Molliex et al., 2015; Nedelsky and Taylor, 2022). For example, the same protein may exhibit different structural forms, such as amyloid fibrils, liquid droplets with gel like properties, amorphous aggregates and membrane associated structures, depending on the stage of disease progression and/or the presence of additional factors (Hamad et al., 2021). Elucidating the detailed structures and mechanisms of aberrant phase transitions in RBPs is an active area of research to better understand the underlying molecular processes and to develop potential therapeutic strategies. Despite these challenges, several approaches to modify biomolecular condensate composition, rates of formation or clearance, and biophysical properties are under intense investigation, not only for ALS/FTD-linked RBPs but also targeting intrinsically disordered proteins in Parkinson’s disease (PD), Alzheimer’s disease (AD) and related dementias (Biesaga et al., 2021; Mitrea et al., 2022). In this section, we discuss recent advances in designing small molecules, peptides, and degrader compounds and other molecular approaches to potentially modulate aberrant RNP condensates and inhibit disease-associated protein aggregation (Table 2). Additionally, we explore the challenges and future prospects of utilizing these small molecules and peptides as therapeutic interventions for neurodegenerative diseases associated with RBP aggregation.

**Table 2:** Therapeutic approaches targeting protein aggregation.

Type of therapeutic approach	Targeted protein	Mechanism of action	Reference
Peptides and small molecules			
CP2	Aβ	Binds to Aβ and inhibit aggregation	Hong et al. (2009)
NPT200-11	α-synuclein	Disrupt the aggregation process	Wrasidlo et al. (2016)
AIM4	TDP-43	Disrupt its phase transitioned gel-like properties	Girdhar et al. (2020)
EGCG	α-synuclein	Inhibit α-synuclein aggregation	Ehrnhoefer et al. (2008) and Fernandes et al. (2021)
D-enantiomeric peptides	Amyloid beta (Aβ)	Prevent the formation of toxic oligomers	Rösener et al. (2018)
Peptide 5	α-synuclein	Disrupts formation of α-synuclein oligomers and fibrils	Krone et al. (2008)
Macromolecular crowding			
Ficoll 70	Aβ peptides	Induces liquid–liquid phase separation to prevent Aβ aggregation	Munishkina et al. (2004)
Dextran 70	Aβ peptides	Induces liquid–liquid phase separation to prevent Aβ aggregation	Siddiqui and Naeem (2023)
PEG 3500	Aβ peptides	Induces liquid–liquid phase separation to prevent Aβ aggregation	Siddiqui and Naeem (2023)
Chemical modification			
K_ac-mimic	Aβ peptides	Reduce aggregation	Adhikari et al. (2020)
Mimetics of PTM peptide	Amyloid-β peptide	Reduce aggregation	Ge et al. (2022)
Sequestration			
Small molecule (10074-G5)	Amyloid-β peptide	Prevent sequestering and aggregation	Heller et al. (2020)
Targeting chaperone proteins			
HSP70	α-Synuclein	Clear alpha-synuclein aggregation	Auluck et al. (2002)
Degraders			
PROteolysis TArgeting Chimeras (PROTACs)	α-synuclein	Binds to the target protein and a ubiquitin E3 ligase	Lai and Crews (2017)
Hydrophobic tagging	Tau	–	Gao et al. (2017)
Autophagy targeting chimera (AUTEC)	α-synuclein	Chaperone mediated autophagy (CMA)	Shaltiel-Karyo et al. (2010)

Based on our current understanding of biomolecular condensate formation and regulation, an emerging strategy is to design or screen for therapeutics that modulate the biophysical properties, macromolecular composition, dynamics and/or free energy landscape of condensate assembly/disassembly (Mitrea et al., 2022). Given the difficulties with rational drug design targeting IDRs, a recent study employed a phenotypic high-content screen to identify small molecules that robustly modulate SG assembly and/or promote disassembly. Among the many hits were ~ 20 SG-modulating compounds that contained extended planar moieties with nucleic acid intercalating properties. One of the hits, mitoxantrone, was able to reduce persistent TDP-43 cytoplasmic puncta in iPSC-derived motor neurons, though the exact mechanism of action remains to be clarified (Fang et al., 2019). Additional small molecules have been tested for modulation of TDP-43 LLPS and/or inhibiting its aggregation. For example, AIM4 ([4,5-bis{(N-carboxy methyl imidazolium)methyl}acridine] dibromide), refers to a class of compounds known as imidazolium-tagged acridines. These compounds have demonstrated the ability to impede the formation of pre-formed TDP-43 aggregates in *in vitro* studies (Prasad et al., 2016). Furthermore, follow-up studies suggest that AIM4 can directly bind to TDP-43 (aa: 288–319) and inhibits LLPS and amyloid-like aggregation of mutant TDP-43 A315T (Girdhar et al., 2020). Another study identified bis-ANS, a small molecule that modulates TDP-43 phase separation in a biphasic manner *in vitro;* however, this compound requires further optimization since it was unable to de-condense SGs in cells (Babinchak et al., 2020).

Additional potential approaches include macromolecular crowding and post-translational/chemical modification, to prevent formation of aberrant condensates or protein aggregates. Molecular crowding refers to the phenomenon where high concentrations of macromolecules in a cellular environment can reduce the propensity of proteins to aggregate. The process of molecular crowding can enhance the protein stability, prevent protein misfolding, and inhibit protein aggregation by increasing the thermodynamic stability of proteins and reducing their conformational entropy (Schreck et al., 2020). Several crowding agent molecules have shown promising results in reducing aggregation propensity of Aβ peptides (Munishkina et al., 2004; Siddiqui and Naeem, 2023). Recently, post translational modifications such as lysine acetylation mimic molecules such as K_ac-mimic molecules have been observed to limit the size and growth of Aβ fibrils (Adhikari et al., 2020).

### Modulation of protein aggregation with small molecules, peptides, and chaperones

Another potential strategy is to identify compounds that can modulate or prevent various steps of fibril formation, regardless of whether fibrillation occurs directly or via an aberrant condensate state (Vendruscolo and Fuxreiter, 2022). Numerous therapeutic approaches that aim to disrupt the aggregation process and mitigate the detrimental effects associated with aggregate formation have been investigated for the treatment of PD (Troncoso-Escudero et al., 2020). The recognition of α-synuclein accumulation as a hallmark of PD has accelerated the development of pharmacological strategies that specifically address misfolded α-synuclein to prevent its aggregation. NPT200-11, a small molecule is designed to specifically target crucial regions of the α-synuclein protein involved in aggregate formation. Through its unique molecular structure, NPT200-11 utilizes its influence on the aggregation process, potentially preventing the formation of toxic oligomers (Wrasidlo et al., 2016). In the transgenic mouse model of PD, oral administration of NPT200-11 demonstrated its capability to penetrate the blood–brain barrier and exert beneficial effects on neuropathological and behavioral outcomes by reducing α-synuclein aggregation (Price et al., 2018). However, additional validation is necessary to establish the safety and effectiveness of NPT200-11. Notably, UCB0599, which is the R-enantiomer of NPT200-11, has demonstrated a favorable safety and tolerability profile in PD patients (Smit et al., 2022). Similarly, several protein structure based small molecules have been discovered to inhibit the formation of Aβ fibrils in the context of AD. For example, the small molecule (10074-G5) stabilizes the soluble state of Aβ and inhibits nucleation steps in the aggregation process (Heller et al., 2020). Tricyclic pyrone molecules (CP2 and TP3) (Hong et al., 2009), beta sheet breaker peptide (Jani et al., 2021), D-enantiomeric peptide (Rösener et al., 2018) and others (Seidler et al., 2022; Murray et al., 2023), also have been highlighted as therapeutic options to prevent the formation of Aβ fibrils.

Likewise, chaperone proteins could be employed to bind unfolded or misfolded proteins and facilitate their proper folding and/or to stabilize native protein conformations, in turn preventing protein aggregation. Additionally, chaperones may bind to early-stage aggregates and inhibit their maturation into fibrils. One well-known chaperone protein is HSP70, which can recognize and bind to hydrophobic regions of proteins, preventing them from forming toxic aggregates in major neurodegenerative diseases such as AD and PD (Auluck et al., 2002; Gifondorwa et al., 2007; Lindstedt et al., 2019).

### Degraders: clearance of protein aggregates

Targeted protein degradation (TPD) has emerged as a promising area of research for modulating proteins that are difficult to target with traditional small molecule inhibitors, particularly those implicated in neurodegenerative diseases. The hotspot regions of RBPs often have broad, shallow active sites or smooth surfaces with few binding sites for small molecules. Therefore, small molecules lose their efficiency as a potential therapeutic option. To address these challenges, Crews and Deshaies developed PROteolysis TArgeting Chimeras (PROTACs) in 2001. PROTACs are hetero bi-functional compounds that consist of a ligand that binds to the target protein and a ubiquitin E3 ligase recruiting group that brings the target protein and the ubiquitin E3 ligase closer together. This proximity facilitates effective degradation of the target protein by the proteasomal machinery (Lai and Crews, 2017). PROTACs have received significant interest in neurodegenerative diseases as a potential therapeutic strategy due to their ability to cross the blood–brain barrier and their ease of administration via various routes, including oral, intravenous, subcutaneous, and intrathecal routes. Arvinas Biopharmaceuticals, Pfizer, and Genentech have all investigated the application of PROTACs in neurodegenerative diseases, particularly in targeting pathological proteins such as tau and α-synuclein in AD and PD. Pioneering research in the use of PROTACs for AD demonstrated the activity of TH006 against the elimination of Tau by recruiting von Hippel–Lindau (VHL) E3 ligase (Ramsay and Di Giovanni, 2017). Since then, different groups have developed Tau-Keap1-CPP, QC-01–175, and C004019 PROTACs employing Keap1, CRBN, and VHL E3 ligases, respectively, for the targeted degradation of Tau in AD models (Cummings, 2021; Karlawish and Grill, 2021; Wang et al., 2021). Small molecule PROTACs have also been shown to induce positive cross-interaction of neuroprotective proteins with toxic proteins, such as transthyretin (a transport protein) with toxic β-amyloids, preventing their oligomerization and subsequent aggregation. Furthermore, small molecule PROTACs that target both Tau and/or α-Synuclein were recently described. This strategy employs Cereblon (CRBN) and VHL E3 ligases with different linkage lengths to design PROTAC compounds against the Tau protein (Kargbo, 2019, 2020). These developments demonstrate the potential of PROTAC technology for targeting “undruggable” targets in neurodegenerative diseases.

An alternative approach for targeting Tau involves the utilization of passively transferred antibodies that bind and degrade tau assemblies (Mukadam et al., 2023). Notably, Mukadam and colleagues showed that Tau-antibody complexes were internalized into the cytosol of neurons, leading to engagement of the cytosolic antibody receptor and E3 ligase TRIM21 and degradation via the ubiquitin-proteasome system. This TRIM21-dependent mechanism was remarkably effective in protecting against seeded aggregation of tau. These findings underscore the significance of the cytosolic compartment as a potential site for immunotherapeutic protection, which holds promise for the development of antibody-based therapies in the treatment of neurodegenerative diseases.

## Concluding remarks

Concentrated efforts by many investigators have highlighted LLPS as an important mechanism for organization of cellular compartments, formation of RNP condensates enriched in RNA and RBPs, and higher order assemblies of condensates with membrane-bound organelles. While a large body of evidence suggests ALS/FTD-linked mutations and associated aberrant RNP condensates have detrimental functional consequences for mRNA stability, transport, and translation in neurons, it is not clear that pathologic aggregation of TDP-43 and other RBPs in ALS/FTD necessarily requires aberrant condensate formation. Rather, it is likely that aberrant LLPS as well as several “hits’” to cellular protein and organelle quality control mechanisms lead to multiple, non-mutually exclusive aggregation pathways. Future work and challenges lie in elucidating effective therapeutic targets shared across familial and sporadic forms of ALS/FTD.

## Author contributions

PG provided the scope and template of the work, and reviewed, edited and wrote sections of the manuscript. AdN and SV wrote the manuscript and reviewed each section. AsN wrote the manuscript, and collected and prepared information for the tables. MS wrote the manuscript, collected and prepared information for the tables, and prepared the schematic figure. All authors contributed to the article and approved the submitted version.

## Funding

This research was supported by the National Institute of Neurological Disorders and Stroke/NIH under Awards R01NS122907 (to PG).

## Conflict of interest

The authors declare that the research was conducted in the absence of any commercial or financial relationships that could be construed as a potential conflict of interest.

## Publisher’s note

All claims expressed in this article are solely those of the authors and do not necessarily represent those of their affiliated organizations, or those of the publisher, the editors and the reviewers. Any product that may be evaluated in this article, or claim that may be made by its manufacturer, is not guaranteed or endorsed by the publisher.
